# Erratum: Fernandez-Palomo, C., et al. Animal Models in Microbeam Radiation Therapy: A Scoping Review. *Cancers* 2020, *12*, 527

**DOI:** 10.3390/cancers12113174

**Published:** 2020-10-29

**Authors:** Cristian Fernandez-Palomo, Jennifer Fazzari, Verdiana Trappetti, Lloyd Smyth, Heidrun Janka, Jean Laissue, Valentin Djonov

**Affiliations:** 1 Institute of Anatomy, University of Bern, 3012 Bern, Switzerland; cristian.fernandez@ana.unibe.ch (C.F.-P.); jennifer.fazzari@ana.unibe.ch (J.F.); verdiana.trappetti@ana.unibe.ch (V.T.); jean-albert.laissue@pathology.unibe.ch (J.L.); 2 Department of Obstetrics & Gynaecology, University of Melbourne, 3057 Parkville, Australia; lloyd.smyth@unimelb.edu.au; 3 Medical Library, University Library Bern, University of Bern, 3012 Bern, Switzerland; heidrun.janka@ub.unibe.ch

The authors wish to make the following corrections to this paper [1]:

The 33 references in Table 1 were incorrectly linked to the bibliography in the original manuscript due to a problem with the reference manager software. The original Table 1 is listed below:

And should be replaced with the following version:

**Table 1:** MRT parameters used in cancer models.

Animal	Cancer Type	Number of Arrays	Microbeam		Peak Dose (Gy)	Valley Dose (Gy)	Evaluated Criteria
			Width (µm)	Spacing (µm)			
Rat [32]	Gliosarcoma (9L)	1 and 2	50	200	400 entrance dose; 350 @ 1 cm depth	12.5 @ 1 cm depth	Animal survival, tumour growth, tumour vasculature, and cell proliferation
Rat [37]	Gliosarcoma (9L)	2	50	200	480 entrance dose; 418 @ 1 cm depth	18.6 @ 1 cm depth	Animal survival, cell cycle, and DNA distribution patterns
Rat [33]	Gliosarcoma (9L)	2	50	200	400 entrance dose; 350 @ 1 cm depth	12.5 @ 1 cm depth	Tumour vasculature and tumour oxygenation
Rat [29]	Gliosarcoma (9L)	1	50	200	400 dose @ tumour (i.e., @ 7 mm depth)	18 dose @ tumour (i.e., @ 7 mm depth)	Animal survival and transcriptomics
Rat [30]	Gliosarcoma (9L)	1	50	200	400 dose @ tumour (i.e., @ 7 mm depth)	8 dose @ tumour (i.e., @ 7 mm depth)	Tumour growth, transcriptomics, and histopathology
Rat [31]	Gliosarcoma (9L)	1	50	200	400 dose @ tumour (i.e., @ 7 mm depth)	17.4 dose @ tumour (i.e., @ 7 mm depth)	Animal survival, tumour growth, cell proliferation, and gene expression
					200 dose @ tumour (i.e., @ 7 mm depth)	8.7 dose @ tumour (i.e., @ 7 mm depth)	
Rat [42]	Glioma (F98)	2	50	200	241.4 entrance dose	10.5 @ 9 mm depth	Tumour vasculature and tumour oxygenation
Mouse [50]	Mammary (EMT6.5/67NR)	1	25	200	560 entrance dose	8.5 @ centre of brain	Animal survival, DNA damage, cell proliferation, and apoptosis
					800 entrance dose	12 @ centre of brain	
		2			280 entrance dose	8.5 @ centre of brain	
					560 entrance dose	17 @ centre of brain	
Rat [15]	Gliosarcoma (9L)	1	27	50	150 entrance dose; 108 @ centre of brain	20 @ centre of brain	Animal survival and histopathology
					250 entrance dose; 179 @ centre of brain	34 @ centre of brain	
					300 entrance dose; 215 @ centre of brain	40 @ centre of brain	
				75	250 entrance dose; 179 @ centre of brain	17 @ centre of brain	
					300 entrance dose; 215 @ centre of brain	20 @ centre of brain	
					500 entrance dose; 359 @ centre of brain	33 @ centre of brain	
				100	500 entrance dose; 359 @ centre of brain	19 @ centre of brain	
Mouse [49]	Mammary (EMT6.5)	1	90	300	800 dose @ tumour	16 dose @ tumour	Tumour ablation
					890 dose @ tumour	18 dose @ tumour	
					970 dose @ tumour	19 dose @ tumour	
					1740 dose @ tumour	35 dose @ tumour	
					1820 dose @ tumour	36 dose @ tumour	
					1900 dose @ tumour	38 dose @ tumour	
		2	90	300	410 dose @ tumour	16 dose @ tumour	
					520 dose @ tumour	21 dose @ tumour	
					650 dose @ tumour	26 dose @ tumour	
Rat [19]	Glioma (C6)	1	25	200	17.5, 35, 70, 350 entrance dose	0.51, 1.03, 2, 10.3	Bystander effects in vivo by clonogenic cell survival
Rat [44]	Glioma (C6)	1	25	200	35, 70, 350 entrance dose	NR	DNA damage
Rat [43]	Glioma (F98)	1	25	200	20, 200 entrance dose	NR	Bystander effects in vivo by clonogenic cell survival and cellular calcium fluxes
Mouse nude [47]	Glioma (F98)		50	400	22, 110 entrance dose	0.5, 2.5	Bystander effects in vivo by clonogenic cell survival and cellular calcium fluxes
Mouse [55]	Mammary (4T1)	1	50	200	150 @ 5 mm depth	7.5 in a 10-mm solid water phantom	Tumour growth, tumour vasculature, and tumour hypoxia
Mouse [52]	Mammary (EMT6.5)	1	25	200	112, 560	NR	Immune response by gene expression and histopathology
Rat [38]	Gliosarcoma (9L)	1	50	200	400 entrance dose	NR	Tumour vasculature, and tumour hypoxia
Rat [25]	Gliosarcoma (9L)	1	25	100	625 entrance dose	NR	Animal survival, tumour growth, and histopathology
Mouse [56]	Squamous cell carcinoma (SCCVII)	1	35	200	442, 625, 884 entrance dose	NR	Animal survival, tumour growth, and tumour ablation
			70	200	442 entrance dose		
Rat [45]	Glioma (C6)	2	25	200	350 entrance dose	NR	Optic nerve damage by histopathology
Mouse [7]	Melanoma (B16F10)	1	50	200	407.6 dose @ tumour	6.2 dose @ tumour	Tumour growth, tumour vasculature, cell proliferation, cell senescence, and immune response
Rat [20]	Gliosarcoma (9L)	1	25	200	625 entrance dose	12.1 dose @ tumour	Animal survival, tumour growth, and histopathology
				100	625 entrance dose	36 dose @ tumour	
Rat [36]	Gliosarcoma (9L)	1	25	200	625 entrance dose	NR	Animal survival, tumour growth, and histopathology
Mouse [54]	Mammary (EMT6.5)	1	25	200	560 entrance dose	11	Biochemical changes by synchrotron Fourier-transform infrared microspectroscopy
Rat [40]	Glioma (C6, F98)	2	25	211	350 entrance dose	NR	Animal survival and object recognition
Rat [41]	Glioma (F98)	2	28	400	350	18 dose @ tumour	Animal survival and cognitive dysfunction
Mouse nude [46]	Gliosarcoma (9L)	1	25	211	500 entrance dose	24 (cross-fired)	Animal survival, tumour growth, and tumour vasculature
Rat [34]	Gliosarcoma (9L)	2	25	211	860 entrance dose	18 @ 1 cm depth	Animal survival and histopathology
			50		480 entrance dose		
			75		320 entrance dose		
Rat [35]	Gliosarcoma (9L)	3	50	211	400, 360 (+24 h), 400 (+48 h) entrance dose	15	Animal survival and histopathology
Rat [39]	Gliosarcoma (9L)	1	27	211	625 entrance dose	NR	Animal survival, histopathology, and immune response
Mouse [51]	Mammary (EMT6.5)	1	25	200	560	11	Transcriptomics
Mouse nude [48]	Glioma (U251)	1	100	500	124	4.8	Tumour growth, histopathology, and apoptosis
		2	20	100	111	8.2	
			100	500	124	9.6	
Mouse [53]	Mammary (EMT6.5)	1	25	200	112, 560	NR	Immune response

NR: not reported.

The authors would like to apologize for any inconvenience caused to the readers by these changes. The original article has been updated.

## Figures and Tables

**Table 1:** MRT Parameters Used in Cancer Models.

Animal	Cancer Type	Number of Arrays	Microbeam		Peak-Dose (Gy)	Valley-Dose (Gy)	Evaluated Criteria
			Width (µm)	Spacing (µm)			
Rat [1]	Gliosarcoma (9L)	1 & 2	50	200	400 entrance dose; 350 @1cm depth	12.5 @1cm depth	Animal survival, tumour growth, tumour vasculature, and cell proliferation
Rat [2]	Gliosarcoma (9L)	2	50	200	480 entrance dose; 418 @1cm depth	18.6 @1cm depth	Animal survival, cell cycle, and DNA distribution patterns
Rat [3]	Gliosarcoma (9L)	2	50	200	400 entrance dose; 350 @1cm depth	12.5 @1cm depth	Tumour vasculature and tumour oxygenation
Rat [4]	Gliosarcoma (9L)	1	50	200	400 dose @tumour (i.e., @7mm depth)	18 dose @tumour (i.e., @7mm depth)	Animal survival and Transcriptomics
Rat [5]	Gliosarcoma (9L)	1	50	200	400 dose @tumour (i.e., @7mm depth)	8 dose @tumour (i.e., @7mm depth)	Tumour growth, transcriptomics, and histopathology
Rat [6]	Gliosarcoma (9L)	1	50	200	400 dose @tumour (i.e., @7mm depth)	17.4 dose @tumour (i.e., @7mm depth)	Animal survival, tumour growth, cell proliferation, and gene expression
					200 dose @tumour (i.e., @7mm depth)	8.7 dose @tumour (i.e., @7mm depth)	
Rat [7]	Glioma (F98)	2	50	200	241.4 entrance dose	10.5 @9mm depth	Tumour vasculature and tumour oxygenation
Mouse [8]	Mammary (EMT6.5/67NR)	1	25	200	560 entrance dose	8.5 @centre of brain	Animal survival, DNA damage, cell proliferation, and apoptosis
					800 entrance dose	12 @centre of brain	
		2			280 entrance dose	8.5 @centre of brain	
					560 entrance dose	17 @centre of brain	
Rat [9]	Gliosarcoma (9L)	1	27	50	150 entrance dose; 108 @centre of brain	20 @centre of brain	Animal survival and histopathology
					250 entrance dose; 179 @centre of brain	34 @centre of brain	
					300 entrance dose; 215 @centre of brain	40 @centre of brain	
				75	250 entrance dose; 179 @centre of brain	17 @centre of brain	
					300 entrance dose; 215 @centre of brain	20 @centre of brain	
					500 entrance dose; 359 @centre of brain	33 @centre of brain	
				100	500 entrance dose; 359 @centre of brain	19 @centre of brain	
Mouse [10]	Mammary (EMT6.5)	1	90	300	800 dose @tumour	16 dose @tumour	Tumour ablation
					890 dose @tumour	18 dose @tumour	
					970 dose @tumour	19 dose @tumour	
					1740 dose @tumour	35 dose @tumour	
					1820 dose @tumour	36 dose @tumour	
					1900 dose @tumour	38 dose @tumour	
		2	90	300	410 dose @tumour	16 dose @tumour	
					520 dose @tumour	21 dose @tumour	
					650 dose @tumour	26 dose @tumour	
Rat [11]	Glioma (C6)	1	25	200	17.5, 35, 70, 350 entrance dose	0.51, 1.03, 2, 10.3	Bystander effects in-vivo by clonogenic cell survival
Rat [12]	Glioma (C6)	1	25	200	35, 70, 350 entrance dose	NR	DNA damage
Rat [13]	Glioma (F98)	1	25	200	20, 200 entrance dose	NR	Bystander effects in-vivo by clonogenic cell survival and cellular calcium fluxes
Mouse nude [14]	Glioma (F98)		50	400	22, 110 entrance dose	0.5, 2.5	Bystander effects in-vivo by clonogenic cell survival and cellular calcium fluxes
Mouse [15]	Mammary (4T1)	1	50	200	150 @5 mm depth	7.5 in a 10 mm solid water phantom	Tumour growth, tumour vasculature, and tumour hypoxia
Mouse [16]	Mammary (EMT6.5)	1	25	200	112, 560	NR	Immune response by gene expression and histopathology
Rat [17]	Gliosarcoma (9L)	1	50	200	400 entrance dose	NR	Tumour vasculature, and tumour hypoxia
Rat [18]	Gliosarcoma (9L)	1	25	100	625 entrance dose	NR	Animal survival, tumour growth, and histopathology
Mouse [19]	Squamous cell carcinoma (SCCVII)	1	35	200	442, 625, 884 entrance dose	NR	Animal survival, tumour growth, and tumour ablation
			70	200	442 entrance dose		
Rat [20]	Glioma (C6)	2	25	200	350 entrance dose	NR	Optic nerve damage by histopathology
Mouse [21]	Melanoma (B16F10)	1	50	200	407.6 dose @tumour	6.2 dose @tumour	Tumour growth, tumour vasculature, cell proliferation, cell senescence, and immune response
Rat [22]	Gliosarcoma (9L)	1	25	200	625 entrance dose	12.1 dose @tumour	Animal survival, tumour growth, and histopathology
				100	625 entrance dose	36 dose @tumour	
Rat [23]	Gliosarcoma (9L)	1	25	200	625 entrance dose	NR	Animal survival, tumour growth, and histopathology
Mouse [24]	Mammary (EMT6.5)	1	25	200	560 entrance dose	11	Biochemical changes by synchrotron Fourier-transform infrared microspectroscopy
Rat [25]	Glioma (C6, F98)	2	25	211	350 entrance dose	NR	Animal survival and object recognition
Rat [26]	Glioma (F98)	2	28	400	350	18 dose @ tumour	Animal survival and cognitive dysfunction
Mouse nude [27]	Gliosarcoma (9L)	1	25	211	500 entrance dose	24 (cross-fired)	Animal survival, tumour growth, and tumour vasculature
Rat [28]	Gliosarcoma (9L)	2	25	211	860 entrance dose	18 @1cm depth	Animal survival and histopathology
			50		480 entrance dose		
			75		320 entrance dose		
Rat [29]	Gliosarcoma (9L)	3	50	211	400, 360 (+24h), 400 (+48h) entrance dose	15	Animal survival and histopathology
Rat [30]	Gliosarcoma (9L)	1	27	211	625 entrance dose	NR	Animal survival, histopathology, and immune response
Mouse [31]	Mammary (EMT6.5)	1	25	200	560	11	Transcriptomics
Mouse nude [32]	Glioma (U251)	1	100	500	124	4.8	Tumour growth, histopathology, and apoptosis
		2	20	100	111	8.2	
			100	500	124	9.6	
Mouse [33]	Mammary (EMT6.5)	1	25	200	112, 560	NR	Immune response

NR: Not-Reported.
