# Supplementary material for: Animal Models in Microbeam Radiation Therapy: A Scoping Review
Source: Cancers (Basel). 2020 Feb 25;12(3):527. doi: 10.3390/cancers12030527 (PMC7139755; doi:10.3390/cancers12030527)
Supplement: Supplementary file 1 [file cancers-12-00527-s001.pdf]

Supplementary Materials: Animal Models in Microbeam Radiation Therapy: A Scoping Review

Cristian Fernandez-Palomo, Jennifer Fazzari, Verdiana Trappetti, Lloyd Smyth, Heidrun Janka, Jean Laissue and Valentin Djonov

A) Electronic search strategies conducted on October 3rd, 2019:

Ovid MEDLINE(R) and Epub Ahead of Print, In-Process & Other Non-Indexed Citations, Daily and Versions(R) <1946 to October 02, 2019>

Search Strategy:

----

1 exp Radiotherapy/ (178770)
2 exp Radiation Tolerance/ (48832)
3 exp Radiation Injuries/ (67796)
4 Technology, Radiologic/ (14225)
5 exp Tomography, X-Ray computed/ (412775)
6 exp X-Rays/ (28187)
7 (radiation effects or radiotherapy).fx. (375879)
8 or/1-7 (905376)
9 Synchrotrons/ (3303)
10 8 and 9 (1263)
11 (radiat* or irradiat* or radiotherap* or radiolog* or x-ray* or x ray* or xray*).ab,kf,ti. (1172914)
12 (microbeam* or micro-beam).ab,kf,ti. (1595)
13 "synchrotron*".ab,kf,ti. (15961)
14 12 or 13 (17234)
15 (therap* or treat* or effect* or outcome*).ab,kf,ti. (11306032)
16 11 and 14 and 15 (3872)

****

Embase (Ovid) <1947 to 2019 October 02>

Search Strategy:

1 exp synchrotron radiation/ (2730)
2 exp synchrotron/ (5593)
3 1 or 2 (8147)
4 radiotherapy/ (134959)
5 cancer radiotherapy/ (188251)
6 exp x-ray tomography/ (45783)
7 X-Ray/ (64437)
8 or/4-7 (415529)
9 3 and 8 (1559)
10 (radiat* or irradiat* or radiotherap* or radiolog* or x-ray* or x ray* or xray*).kw,dq,tw. (1467788)
11 (microbeam* or micro-beam).kw,dq,tw. (1677)
12 (synchrotron or synchrotron radiation).kw,dq,tw. (12249)
13 11 or 12 (13524)
14 (therap* or effect* or treat* or outcome*).kw,tw. (14192639)
15 10 and 13 and 14 (3649)
16 9 or 15 (4625)
17 limit 16 to (embase) (3069)

****

Cochrane Library

Search Strategy:

1 MeSH descriptor: [Radiotherapy] explode all trees (5977)
2 MeSH descriptor: [Radiation Tolerance] explode all trees (651)
3 MeSH descriptor: [Radiation Injuries] explode all trees (1391)
4 MeSH descriptor: [Technology, Radiologic] explode all trees (47)
5 MeSH descriptor: [Tomography, X-Ray Computed] explode all trees (4989)
6 MeSH descriptor: [X-Rays] explode all trees (46)
7 MeSH descriptor: explode all trees and with qualifier(s): [radiation effects - RE] (2898)
8 #1 or #2 or #3 or #4 or #5 or #6 or #7 (13441)
9 MeSH descriptor: [Synchrotrons] explode all trees (2)
10 #8 and #9 (1)
11 (radiat* or irradiat* or radiotherap* or radiolog* or x-ray* or x ray* or xray*):ti,ab,kw (Word variations have been searched) (76228)
12 (microbeam or micro-beam):ti,ab,kw (Word variations have been searched) (21)
13 (synchrotron*):ti,ab,kw (Word variations have been searched) (24)
14 #12 or #13 (44)
15 #11 and #14 (27)
16 #10 or #15 (27)

****
Scopus

Search Strategy:

(TITLE-ABS-KEY (radiat* OR irradiat* OR radiotherap* OR radiolog* OR x-ray* OR x AND ray* OR xray*) AND TITLE-ABS-KEY (microbeam* OR micro-beam* OR synchrotron*) AND TITLE-ABS-KEY (therap* OR effect* OR outcome* OR treat*))

Search results: 380

****
Livivo - ZB MED Search portal

Search Strategy:

(Synchrotron OR Microbeam OR Micro-Beam) AND (radiation OR irradiation OR radiotherapy)

Source excluded: Medline

Search results: 54

****
Clinical Trials.com

Search Strategy:

(synchrotron OR microbeam OR micro-beam) AND (radiation OR irradiation or radiotherapy)

Search results: 4

****
ICTRP Trial Register (WHO)

Search Strategy:

(synchrotron OR microbeam OR micro-beam) AND (radiation OR irradiation or radiotherapy)

Search results: 2

B) List of the 95 Included Studies:

1. Anschel, David J., Alberto Bravin, and Pantaleo Romanelli. 'Microbeam Radiosurgery Using Synchrotron-Generated Submillimetric Beams: A New Tool for the Treatment of Brain Disorders'. Neurosurgical Review 34, no. 2 (April 2011): 133–42. https://doi.org/10.1007/s10143-010-0292-3.
2. Bencokova, Z, J Balosso, and N Foray. 'Radiobiological Features of the Anti-Cancer Strategies Involving Synchrotron X-Rays'. Journal of Synchrotron Radiation 15, no. 1 (2007): 74–85.
3. Billena, Cole, and Atif J Khan. 'A Current Review of Spatial Fractionation: Back to the Future?' International Journal of Radiation Oncology Biology Physics 104, no. 1 (2019): 177–187. https://doi.org/10.1016/j.ijrobp.2019.01.073.
4. Blattmann, H, J Gebbers, E Brauerkrisch, a Bravin, G Leduc, W Burkard, M Dimichiel, V Djonov, D Slatkin, and J Stepanek. 'Applications of Synchrotron X-Rays to Radiotherapy'. Nuclear Instruments and Methods in Physics Research Section A: Accelerators, Spectrometers, Detectors and Associated Equipment 548, no. 1–2 (August 2005): 17–22. https://doi.org/10.1016/j.nima.2005.03.060.
5. Bouchet, A, A Boumendjel, E Khalil, R Serduc, E Brauer, E A Siegbahn, J A Laissue, and J Boutonnat. 'Chalcone JAI-51 Improves Efficacy of Synchrotron Microbeam Radiation Therapy of Brain Tumors'. J Synchrotron Radiat 19, no. Pt 4 (2012): 478–482.
6. Bouchet, A, N Sakakini, M El Atifi, C Le Clec'h, E Brauer, A Moisan, P Deman, P Rihet, G Le Duc, and L Pelletier. 'Early Gene Expression Analysis in 9L Orthotopic Tumor-Bearing Rats Identifies Immune Modulation in Molecular Response to Synchrotron Microbeam Radiation Therapy'. PLoS ONE [Electronic Resource] 8, no. 12 (2013): e81874.
7. Bouchet, A, N Sakakini, M El Atifi, C Le Clec'h, E Bräuer-Krisch, L Rogalev, J Laissue, P Rihet, G Le Duc, and L Pelletier. 'Identification of AREG and PLK1 Pathway Modulation as a Potential Key of the Response of Intracranial 9L Tumor to Microbeam Radiation Therapy'. International Journal of Cancer. Journal International Du Cancer in print (2014).
8. Bouchet, Audrey, Elke Bräuer-Krisch, Yolanda Prezado, Michèle El Atifi, Léonid Rogalev, Céline Le Clec'h, Jean Albert Laissue, Laurent Pelletier, and Géraldine Le Duc. 'Better Efficacy of Synchrotron Spatially Microfractionated Radiation Therapy Than Uniform Radiation Therapy on Glioma'. International Journal of Radiation Oncology Biology Physics 95, no. 5 (2016): 1485–1494. https://doi.org/10.1016/j.ijrobp.2016.03.040.
9. Bouchet, Audrey, Benjamin Lemasson, Thomas Christen, Marine Potez, Claire Rome, Nicolas Coquery, Céline Le Clec'H, et al. 'Synchrotron Microbeam Radiation Therapy Induces Hypoxia in Intracerebral Gliosarcoma but Not in the Normal Brain'. Radiotherapy and Oncology 108, no. 1 (2013): 143–148. https://doi.org/10.1016/j.radonc.2013.05.013.
10. Bouchet, Audrey, Benjamin Lemasson, Géraldine Le Duc, Cécile Maisin, Elke Bräuer-Krisch, Erik Albert Siegbahn, Luc Renaud, et al. 'Preferential Effect of Synchrotron Microbeam Radiation Therapy on Intracerebral 9l Gliosarcoma Vascular Networks'. International Journal of Radiation Oncology Biology Physics 78, no. 5 (2010): 1503–1512. https://doi.org/10.1016/j.ijrobp.2010.06.021.
11. Bouchet, Audrey, Marine Potez, Nicolas Coquery, Claire Rome, Benjamin Lemasson, Elke Bräuer-Krisch, Chantal Rémy, et al. 'Permeability of Brain Tumor Vessels Induced by Uniform or Spatially Microfractionated Synchrotron Radiation Therapies'. International Journal of Radiation Oncology Biology Physics 98, no. 5 (2017): 1174–1182. https://doi.org/10.1016/j.ijrobp.2017.03.025.
12. Bouchet, Audrey, Raphäel Serduc, Jean Albert Laissue, and Valentin Djonov. 'Effects of Microbeam Radiation Therapy on Normal and Tumoral Blood Vessels'. Physica Medica 31, no. 6 (2015): 634–641. https://doi.org/10.1016/j.ejmp.2015.04.014.
13. Bräuer-Krisch, E., H. Requardt, P. Régnard, S. Corde, E. Siegbahn, G. LeDuc, T. Brochard, H. Blattmann, J. Laissue, and A. Bravin. 'New Irradiation Geometry for Microbeam Radiation Therapy'. Physics in Medicine and Biology 50, no. 13 (2005): 3103–3111. https://doi.org/10.1088/0031-9155/50/13/009.
14. Bräuer-Krisch, E., R Serduc, E A Siegbahn, G Le Duc, Y Prezado, A Bravin, H Blattmann, and J A Laissue. 'Effects of Pulsed, Spatially Fractionated, Microscopic Synchrotron X-Ray Beams on Normal and Tumoral Brain Tissue'. Mutation Research - Reviews in Mutation Research 704, no. 1–3 (June 2010): 160–166. https://doi.org/10.1016/j.mrrev.2009.12.003.

15. Bravin, Alberto, Pawel Olko, Elisabeth Schültke, and Jan J. Wilkens. 'SYRA3 COST Action - Microbeam Radiation Therapy: Roots and Prospects'. Physica Medica 31, no. 6 (2015): 561–563. https://doi.org/10.1016/j.ejmp.2015.06.002.
16. Brönnimann, Daniel, Audrey Bouchet, Christoph Schneider, Marine Potez, Raphaël Serduc, Elke Bräuer-Krisch, Werner Graber, Stephan Von Gunten, Jean Albert Laissue, and Valentin Djonov. 'Synchrotron Microbeam Irradiation Induces Neutrophil Infiltration, Thrombocyte Attachment and Selective Vascular Damage in Vivo'. Scientific Reports 6 (2016): 33601. https://doi.org/10.1038/srep33601.
17. Crosbie, Jeffrey C., Robin L. Anderson, Kai Rothkamm, Christina M. Restall, Leonie Cann, Saleela Ruwanpura, Sarah Meachem, et al. 'Tumor Cell Response to Synchrotron Microbeam Radiation Therapy Differs Markedly from Cells in Normal Tissues'. International Journal of Radiation Oncology, Biology, Physics 77, no. 3 (2010): 886–894. https://doi.org/10.1016/j.ijrobp.2010.01.035.
18. Dilmanian, F A, J Kalef-Ezra, M J Petersen, G Bozios, J Vosswinkel, F Giron, B Ren, R Yakupov, and G Antonakopoulos. 'Could X-Ray Microbeams Inhibit Angioplasty-Induced Restenosis in the Rat Carotid Artery?' Cardiovascular Radiation Medicine 4, no. 3 (September 2003): 139–145. https://doi.org/10.1016/S1522-1865(03)00180-X.
19. Dilmanian, F A, G M Morris, G Le Duc, X Huang, B Ren, T Bacarian, J C Allen, et al. 'Response of Avian Embryonic Brain to Spatially Segmented X-Ray Microbeams.' Cellular and Molecular Biology (Noisy-Le-Grand, France) 47, no. 3 (May 2001): 485–93.
20. Dilmanian, F a, Y Qu, S Liu, C D Cool, J Gilbert, J F Hainfeld, C a Kruse, et al. 'X-Ray Microbeams: Tumor Therapy and Central Nervous System Research.' Nuclear Instruments & Methods in Physics Research. Section A, Accelerators, Spectrometers, Detectors and Associated Equipment 548, no. 1–2 (August 2005): 30–37. https://doi.org/10.1016/j.nima.2005.03.062.
21. Dilmanian, F. Avraham, Terry M. Button, Géraldine Le Duc, Nan Zhong, Louis A. Peña, Jennifer A. L. Smith, Steve R. Martinez, et al. 'Response of Rat Intracranial 9L Gliosarcoma to Microbeam Radiation Therapy'. Neuro-Oncology 4, no. 1 (2002): 26–38. https://doi.org/10.1093/neuonc/4.1.26.
22. Dilmanian, F. Avraham, Gerard M. Morris, Nan Zhong, Tigran Bacarian, James F. Hainfeld, John Kalef-Ezra, Laura J. Brewington, Jennifer Tammam, and Eliot M. Rosen. 'Murine EMT-6 Carcinoma: High Therapeutic Efficacy of Microbeam Radiation Therapy.' Radiation Research 159, no. 5 (May 2003): 632–41. https://doi.org/10.1667/0033-7587(2003)159[0632:mechte]2.0.co;2.
23. Eling, Laura, Audrey Bouchet, Christian Nemoz, Valentin Djonov, Jacques Balosso, Jean Laissue, Elke Bräuer-Krisch, Jean Francois Adam, and Raphael Serduc. 'Ultra High Dose Rate Synchrotron Microbeam Radiation Therapy. Preclinical Evidence in View of a Clinical Transfer'. Radiotherapy and Oncology 139 (2019): 56–61. https://doi.org/10.1016/j.radonc.2019.06.030.
24. Fardone, Erminia, Alberto Bravin, Alfredo Conti, Elke Bräuer-Krisch, Herwig Requardt, Domenico Bucci, Geraldine Le Duc, Giuseppe Battaglia, and Pantaleo Romanelli. 'Rat Sensorimotor Cortex Tolerance to Parallel Transections Induced by Synchrotron-Generated X-Ray Microbeams'. Scientific Reports 7, no. 1 (2017): 14290. https://doi.org/10.1038/s41598-017-14757-3.
25. Fardone, Erminia, Benoît Pouyatos, Elke Bräuer-Krisch, Stefan Bartzsch, Hervè Mathieu, Herwig Requardt, Domenico Bucci, et al. 'Synchrotron-Generated Microbeams Induce Hippocampal Transections in Rats'. Scientific Reports 8, no. 1 (2018): 184. https://doi.org/10.1038/s41598-017-18000-x.
26. Fernandez-Palomo, C, C Mothersill, J Laissue, C Seymour, E Schültke, C Seymour, and E Schültke. 'γ-H2AX as a Marker for Dose Deposition in the Brain of Wistar Rats after Synchrotron Microbeam Radiation'. PLoS ONE 10, no. 3 (2015). https://www2.scopus.com/inward/record.uri?eid=2-s2.0-84925855885&doi=10.1371%2Fjournal.pone.0119924&partnerID=40&md5=a648c32ee033c4f6d863e499b4b90fdb.
27. Fernandez-Palomo, Cristian, Elke Bräuer-Krisch, Jean Laissue, Dusan Vukmirovic, Hans Blattmann, Colin Seymour, Elisabeth Schültke, and Carmel Mothersill. 'Use of Synchrotron Medical Microbeam Irradiation to Investigate Radiation-Induced Bystander and Abscopal Effects in Vivo'. Physica Medica 31, no. 6 (2015): 584–595. https://doi.org/10.1016/j.ejmp.2015.03.004.
28. Fernandez-Palomo, Cristian, Elisabeth Schültke, Elke Bräuer-Krisch, Jean Albert Laissue, Hans Blattmann, Colin Seymour, and Carmel Mothersill. 'Investigation of Abscopal and Bystander Effects in Immunocompromised Mice after Exposure to Pencilbeam and Microbeam Synchrotron Radiation'. Health Physics 111, no. 2 (2016): 149–159. https://doi.org/10.1097/HP.0000000000000525.
29. Fernandez-Palomo, Cristian, Elisabeth Schültke, Richard Smith, Elke Bräuer-Krisch, Jean Laissue, Christian Schroll, Jennifer Fazzari, Colin Seymour, and Carmel Mothersill. 'Bystander Effects in Tumor-Free and Tumor-Bearing Rat Brains Following Irradiation by Synchrotron X-Rays'. International Journal of Radiation Biology 89, no. 6 (2013): 445–453. https://doi.org/10.3109/09553002.2013.766770.

30. Fu, S, and G Jiang. 'Potential Application of SSRF in Radiation Oncology: The Aspects of Radiobiology'. Nuclear Science and Techniques 20, no. 6 (2009): 325–330.
31. Fukunaga, Hisanori, Kiichi Kaminaga, Takuya Sato, Karl T Butterworth, Ritsuko Watanabe, Noriko Usami, Takehiko Ogawa, Akinari Yokoya, and Kevin M Prise. 'High-Precision Microbeam Radiotherapy Reveals Testicular Tissue-Sparing Effects for Male Fertility Preservation'. Scientific Reports 9, no. 1 (2019): 12618. https://doi.org/10.1038/s41598-019-48772-3.
32. Ghita, Mihaela, Cristian Fernandez-Palomo, Hisanori Fukunaga, Pil M Fredericia, Giuseppe Schettino, Elke Bräuer-Krisch, Karl T Butterworth, Stephen J McMahon, and Kevin M Prise. 'Microbeam Evolution: From Single Cell Irradiation to Pre-Clinical Studies'. International Journal of Radiation Biology 94, no. 8 (2018): 708–718. https://doi.org/10.1080/09553002.2018.1425807.
33. Griffin, Robert J, Nathan A Koonce, Ruud P. M Dings, Eric Siegel, Eduardo G Moros, Elke Bräuer-Krisch, and Peter M Corry. 'Microbeam Radiation Therapy Alters Vascular Architecture and Tumor Oxygenation and Is Enhanced by a Galectin-1 Targeted Anti-Angiogenic Peptide'. Radiation Research 177, no. 6 (2012): 804–812. https://doi.org/10.1667/rr2784.1.
34. Grotzer, M.A., E. Schültke, E. Bräuer-Krisch, and J.A. Laissue. 'Microbeam Radiation Therapy: Clinical Perspectives'. Physica Medica 31, no. 6 (September 2015): 564–567. https://doi.org/10.1016/j.ejmp.2015.02.011.
35. Ibahim, M J, Y Yang, J C Crosbie, A Stevenson, L Cann, P Paiva, and P A Rogers. 'Eosinophil-Associated Gene Pathways but Not Eosinophil Numbers Are Differentially Regulated between Synchrotron Microbeam Radiation Treatment and Synchrotron Broad-Beam Treatment by 48 Hours Postirradiation'. Radiation Research 185, no. 1 (2015): 60. https://doi.org/10.1667/rr14115.1.
36. Ishii, Taiji, Takehiko Ueyama, Michiko Shigyo, Masaaki Kohta, Takeshi Kondoh, Tomoharu Kuboyama, Tatsuya Uebi, et al. 'A Novel Rac1-GSPT1 Signaling Pathway Controls Astrogliosis Following Central Nervous System Injury'. Journal of Biological Chemistry 292, no. 4 (2017): 1240–1250. https://doi.org/10.1074/jbc.M116.748871.
37. Kennedy, Charles, and Avraham Dilmanian. 'Prospects for Microbeam Radiation Therapy of Brain Tumours in Children'. Developmental Medicine & Child Neurology 49, no. 8 (August 2007): 566–566. https://doi.org/10.1111/j.1469-8749.2007.00566.x.
38. Laissue, J A, H Blattmann, H P Wagner, M A Grotzer, and D N Slatkin. 'Prospects for Microbeam Radiation Therapy of Brain Tumours in Children to Reduce Neurological Sequelae.' Developmental Medicine and Child Neurology 49, no. 8 (August 2007): 577–81. https://doi.org/10.1111/j.1469-8749.2007.00577.x.
39. Laissue, J. A., G. Geiser, P. O. Spanne, F. A. Dilmanian, J. O. Gebbers, M. Geiser, X. Y. Wu, et al. 'Neuropathology of Ablation of Rat Gliosarcomas and Contiguous Brain Tissues Using a Microplanar Beam of Synchrotron-Wiggler-Generated X Rays'. International Journal of Cancer 78, no. 5 (23 November 1998): 654–60.
40. Laissue, Jean A., Stefan Bartzsch, Hans Blattmann, Elke Bräuer-Krisch, Alberto Bravin, Dominique Dalléry, Valentin Djonov, et al. 'Response of the Rat Spinal Cord to X-Ray Microbeams'. Radiotherapy and Oncology 106, no. 1 (2013): 106–111. https://doi.org/10.1016/j.radonc.2012.12.007.
41. Laissue, Jean A., Hans Blattmann, Marco Di Michiel, Daniel N. Slatkin M.d, Nadia Lyubimova, Raphael Guzman, Werner Zimmermann, et al. 'Weanling Piglet Cerebellum: A Surrogate for Tolerance to MRT (Microbeam Radiation Therapy) in Pediatric Neuro-Oncology'. In Penetrating Radiation Systems and Applications III, 4508:65–73. International Society for Optics and Photonics, 2001. https://doi.org/10.1117/12.450774.
42. Laissue, Jean A., Hans Blattmann, Erik A. Siegbahn, and Daniel N. Slatkin. 'A Misprint in a Description of Microbeam Irradiations of Rats' Heads'. Veterinary Ophthalmology 15, no. 3 (May 2012): 210–11. https://doi.org/10.1111/j.1463-5224.2012.01023.x.
43. Laissue, Jean A., Nadia Lyubimova, Hans-Peter Wagner, David W. Archer, Daniel N. Slatkin, Marco Di Michiel, Christian Nemoz, et al. 'Microbeam Radiation Therapy'. In Medical Applications of Penetrating Radiation, edited by H. Bradford Barber and Hans Roehrig, 3770:38. International Society for Optics and Photonics, 1999. https://doi.org/10.1117/12.368185.
44. Lemasson, Benjamin, Audrey Bouchet, Cécile Maisin, Thomas Christen, Géraldine Le Duc, Chantal Rémy, Emmanuel L Barbier, and Raphaël Serduc. 'Multiparametric MRI as an Early Biomarker of Individual Therapy Effects during Concomitant Treatment of Brain Tumours'. NMR in Biomedicine 28, no. 9 (2015): 1163–1173. https://doi.org/10.1002/nbm.3357.
45. Livingstone, Jayde, Jean François Adam, Jeffrey C. Crosbie, Chris J. Hall, Jessica E. Lye, Jonathan McKinlay, Daniele Pelliccia, et al. 'Preclinical Radiotherapy at the Australian Synchrotron's Imaging and Medical Beamline: Instrumentation, Dosimetry and a Small-Animal Feasibility Study'. Journal of Synchrotron Radiation 24, no. 4 (2017): 854–865. https://doi.org/10.1107/S1600577517006233.

46. Lobachevsky, Pavel N., Jessica Ventura, Giannak, L Ropoulou, Helen Forrester, Jason S. Palazzolo, Nicole M. Haynes, et al. 'A Functional Immune System Is Required for the Systemic Genotoxic Effects of Localized Irradiation'. International Journal of Radiation Oncology, Biology, Physics 103, no. 5 (2019): 1184–1193. https://doi.org/10.1016/j.ijrobp.2018.11.066.
47. MATSUMOTO, Hideki, Masanori TOMITA, Kensuke OTSUKA, and Masanori HATASHITA. 'A New Paradigm in Radioadaptive Response Developing from Microbeam Research'. Journal of Radiation Research 50, no. Suppl.A (2009): A67–A79. https://doi.org/10.1269/jrr.09003s.
48. Miura, M, H Blattmann, E Bräuer-Krisch, A Bravin, A L Hanson, M M Nawrocky, P L Micca, D N Slatkin, and J A Laissue. 'Radiosurgical Palliation of Aggressive Murine SCCVII Squamous Cell Carcinomas Using Synchrotron-Generated X-Ray Microbeams'. The British Journal of Radiology 79, no. 937 (January 2006): 71–75. https://doi.org/10.1259/bjr/50464795.
49. Mohamed, A, S Worobec, and E Schultke. 'Assessment of Rat Optic Nerve Damage Due to Microbeam Radiation Therapy in the Treatment of Glioblastomas'. Biomedical Sciences Instrumentation 44 (2008): 501–506.
50. Mothersill, Carmel, Cristian Fernandez-Palomo, Jennifer Fazzari, Richard Smith, Elisabeth Schültke, Elke Bräuer-Krisch, Jean Laissue, Christian Schroll, and Colin Seymour. 'Transmission of Signals from Rats Receiving High Doses of Microbeam Radiation to Cage Mates: An Inter-Mammal Bystander Effect'. Dose-Response 12, no. 1 (2014): 72–92. https://doi.org/10.2203/dose-response.13-011.Mothersill.
51. Mukumoto, Naritoshi, Masao Nakayama, Hiroaki Akasaka, Yasuyuki Shimizu, Saki Osuga, Daisuke Miyawaki, Kenji Yoshida, et al. 'Sparing of Tissue by Using Micro-Slit-Beam Radiation Therapy Reduces Neurotoxicity Compared with Broad-Beam Radiation Therapy'. Journal of Radiation Research 58, no. 1 (2017): 17–23. https://doi.org/10.1093/jrr/rrw065.
52. Potez, Marine, Audrey Bouchet, Jeannine Wagner, Mattia Donzelli, Elke Bräuer-Krisch, John W Hopewell, Jean Laissue, and Valentin Djonov. 'Effects of Synchrotron X-Ray Micro-Beam Irradiation on Normal Mouse Ear Pinnae'. International Journal of Radiation Oncology Biology Physics 101, no. 3 (2018): 680–689. https://doi.org/10.1016/j.ijrobp.2018.02.007.
53. Potez, Marine, Cristian Fernandez-Palomo, Audrey Bouchet, Verdiana Trappetti, Mattia Donzelli, Michael Krisch, Jean Laissue, Vladislav Volarevic, and Valentin Djonov. 'Synchrotron Microbeam Radiation Therapy as a New Approach for the Treatment of Radioresistant Melanoma: Potential Underlying Mechanisms'. International Journal of Radiation Oncology Biology Physics 105, no. 5 (2019): 1126–1136. https://doi.org/10.1016/j.ijrobp.2019.08.027.
54. Pouyatos, B, C Nemoz, T Chabrol, M Potez, E. Bräuer, L Renaud, K Pernet-Gallay, et al. 'Synchrotron X-Ray Microtransections: A Non Invasive Approach for Epileptic Seizures Arising from Eloquent Cortical Areas'. Scientific Reports 6 (2016): 27250. https://doi.org/10.1038/srep27250.
55. Pouyatos, Benoît, Raphaël Serduc, Mathilde Chipaux, Tanguy Chabrol, E Brauer-Krisch, Christian Nemoz, Hervé Mathieu, et al. 'Synchrotron X-Ray Interlaced Microbeams Suppress Paroxysmal Oscillations in Neuronal Networks Initiating Generalized Epilepsy'. Neurobiology of Disease 51 (2013): 152–160. https://doi.org/10.1016/j.nbd.2012.11.005.
56. Priyadarshika, R C U, J C Crosbie, B Kumar, and P a W Rogers. 'Biodosimetric Quantification of Short-Term Synchrotron Microbeam versus Broad-Beam Radiation Damage to Mouse Skin Using a Dermatopathological Scoring System.' The British Journal of Radiology 84, no. 1005 (September 2011): 833–42. https://doi.org/10.1259/bjr/58503354.
57. Régnard, Pierrick, Elke Bräuer-Krisch, Irène Troprès, Jani Keyriläinen, Alberto Bravin, and Géraldine Le Duc. 'Enhancement of Survival of 9L Gliosarcoma Bearing Rats Following Intracerebral Delivery of Drugs in Combination with Microbeam Radiation Therapy'. European Journal of Radiology 68, no. 3 SUPPL. (2008): S151–S155. https://doi.org/10.1016/j.ejrad.2008.04.049.
58. Régnard, Pierrick, Géraldine Le Duc, Elke Bräuer-Krisch, Irène Troprès, Erik Albert Siegbahn, Audrey Kusak, Charlotte Clair, et al. 'Irradiation of Intracerebral 9L Gliosarcoma by a Single Array of Microplanar X-Ray Beams from a Synchrotron: Balance between Curing and Sparing'. Physics in Medicine and Biology 53, no. 4 (2008): 861–878. https://doi.org/10.1088/0031-9155/53/4/003.
59. Romanelli, P, A Bravin, E Fardone, and G Battaglia. 'New Radiosurgical Paradigms to Treat Epilepsy Using Synchrotron Radiation'. Epilepsy Towards the Next Decade: New Trends and Hopes in Epileptology, 2015, 231–236.
60. Romanelli, P, E Fardone, G Battaglia, E Brauer-Krisch, Y Prezado, H Requardt, G Le Duc, et al. 'Synchrotron-Generated Microbeam Sensorimotor Cortex Transections Induce Seizure Control without Disruption of Neurological Functions'. PLoS ONE [Electronic Resource] 8, no. 1 (2013): e53549.

61. Romanelli, Pantaleo, and Alberto Bravin. 'Synchrotron-Generated Microbeam Radiosurgery: A Novel Experimental Approach to Modulate Brain Function'. Neurological Research 33, no. 8 (2011): 825–831. https://doi.org/10.1179/016164111X13123658647445.
62. Romanelli, Pantaleo, Erminia Fardone, Domenico Bucci, Giuseppe Battaglia, Elke Bräuer-Krisch, Herwig Requardt, Geraldine Le Duc, and Alberto Bravin. 'Microradiosurgical Cortical Transections Generated by Synchrotron Radiation'. Physica Medica 31, no. 6 (2015): 642–646. https://doi.org/10.1016/j.ejmp.2015.05.007.
63. Rothkamm, Kai, Jeffrey C Crosbie, Frances Daley, Sarah Bourne, Paul R Barber, Borivoj Vojnovic, Leonie Cann, and Peter a W Rogers. 'In Situ Biological Dose Mapping Estimates the Radiation Burden Delivered to "spared" Tissue between Synchrotron X-Ray Microbeam Radiotherapy Tracks.' PloS One 7, no. 1 (January 2012): e29853. https://doi.org/10.1371/journal.pone.0029853.
64. Rothman, E.Z., S.L. Hulbert, and N.M. Lazarz. 'National Synchrotron Light Source. [Annual Report], October 1, 1992--September 30, 1993. Subsection B-132: Microplanar Beam Radiotherapy [MRT] of Malignant Brain Tumors In Rats', 1 April 1994. https://doi.org/10.2172/10169934.
65. Sabatasso, Sara, Jean Albert Laissue, Ruslan Hlushchuk, Werner Graber, Alberto Bravin, Elke Bräuer-Krisch, Stéphanie Corde, Hans Blattmann, Guenther Gruber, and Valentin Djonov. 'Microbeam Radiation-Induced Tissue Damage Depends on the Stage of Vascular Maturation'. International Journal of Radiation Oncology Biology Physics 80, no. 5 (2011): 1522–1532. https://doi.org/10.1016/j.ijrobp.2011.03.018.
66. Sandmeyer, Lynne S., Amber Sheikh, Elisabeth Schültke, Daryl Fourney, and Bruce H. Grahn. 'Chronic Ocular Lesions Associated with Bi-Directional Microbeam Radiation Therapy in an Experimental Rat Study for Therapy of C6 and F98 Gliomas'. Veterinary Ophthalmology 11, no. 5 (September 2008): 290–98. https://doi.org/10.1111/j.1463-5224.2008.00649.x.
67. Schültke, E., E. Bräuer-Krisch, H Blattmann, H Requardt, J A Laissue, and G. Hildebrandt. 'Survival of Rats Bearing Advanced Intracerebral F 98 Tumors after Glutathione Depletion and Microbeam Radiation Therapy: Conclusions from a Pilot Project'. Radiation Oncology 13, no. 1 (2018): 89. https://doi.org/10.1186/s13014-018-1038-6.
68. Schültke, Elisabeth, Jacques Balosso, Thomas Breslin, Guido Cavaletti, Valentin Djonov, Francois Esteve, Michael Grotzer, Guido Hildebrandt, Alexander Valdman, and Jean Laissue. 'Microbeam Radiation Therapy - Grid Therapy and beyond: A Clinical Perspective'. British Journal of Radiology 90, no. 1078 (2017): 20170073. https://doi.org/10.1259/bjr.20170073.
69. Schültke, Elisabeth, Bernhard H.J. Juurlink, Khalid Ataelmannan, Jean Laissue, Hans Blattmann, Elke Bräuer-Krisch, Alberto Bravin, et al. 'Memory and Survival after Microbeam Radiation Therapy'. European Journal of Radiology 68, no. 3 SUPPL. (2008): S142–S146. https://doi.org/10.1016/j.ejrad.2008.04.051.
70. Schweizer, P M, P Spanne, M Di Michiel, U Jauch, H Blattmann, and J A Laissue. 'Tissue Lesions Caused by Microplanar Beams of Synchrotron-Generated X-Rays in Drosophila Melanogaster'. International Journal of Radiation Biology 76, no. 4 (2000): 567–574. https://doi.org/10.1080/095530000138583.
71. Serduc, Raphaël, Audrey Bouchet, Elke Bräuer-Krisch, Jean A Laissue, Jenny Spiga, Sukhéna Sarun, Alberto Bravin, et al. 'Synchrotron Microbeam Radiation Therapy for Rat Brain Tumor Palliation - Influence of the Microbeam Width at Constant Valley Dose'. Physics in Medicine and Biology 54, no. 21 (2009): 6711–6724. https://doi.org/10.1088/0031-9155/54/21/017.
72. Serduc, Raphaël, E Brauer-Krisch, Erik a Siegbahn, Audrey Bouchet, Benoit Pouyatos, Romain Carron, Nicolas Pannetier, et al. 'High-Precision Radiosurgical Dose Delivery by Interlaced Microbeam Arrays of High-Flux Low-Energy Synchrotron X-Rays.' PloS One 5, no. 2 (January 2010): e9028. https://doi.org/10.1371/journal.pone.0009028.
73. Serduc, Raphaël, Elke Bräuer-Krisch, Audrey Bouchet, Luc Renaud, Thierry Brochard, Alberto Bravin, Jean Albert Laissue, and Géraldine Le Duc. 'First Trial of Spatial and Temporal Fractionations of the Delivered Dose Using Synchrotron Microbeam Radiation Therapy'. Journal of Synchrotron Radiation 16, no. 4 (July 2009): 587–590. https://doi.org/10.1107/S0909049509012485.
74. Serduc, Raphael, Thomas Christen, Jean Albert Laissue, Regine Régine Farion, Audrey Bouchet, Boudewijn van der Sanden, Christoph Segebarth, et al. 'Brain Tumor Vessel Response to Synchrotron Microbeam Radiation Therapy: A Short-Term in Vivo Study.' Physics in Medicine and Biology 53, no. 13 (July 2008): 3609–22. https://doi.org/10.1088/0031-9155/53/13/015.
75. Serduc, Raphaël, Yohan Van De Looij, Gilles Francony, Olivier Verdonck, Boudewijn Van Der Sanden, Jean Laissue, Régine Farion, et al. 'Characterization and Quantification of Cerebral Edema Induced by Synchrotron X-Ray Microbeam Radiation Therapy'. Physics in Medicine and Biology 53, no. 5 (2008): 1153–1166. https://doi.org/10.1088/0031-9155/53/5/001.
76. Serduc, Raphaël, Pascale Vérant, Jean Claude Vial, Régine Farion, Linda Rocas, Chantal Rémy, Taoufik Fadlallah, et al. 'In Vivo Two-Photon Microscopy Study of Short-Term Effects of Microbeam Irradiation on

Normal Mouse Brain Microvasculature'. International Journal of Radiation Oncology Biology Physics 64, no. 5 (2006): 1519–1527. https://doi.org/10.1016/j.ijrobp.2005.11.047.
77. Sharma, Monica, Jeffrey C Crosbie, Ljiljana Puskar, and Peter A.W. Rogers. 'Microbeam-Irradiated Tumour Tissue Possesses a Different Infrared Absorbance Profile Compared to Broad Beam and Sham-Irradiated Tissue'. International Journal of Radiation Biology 89, no. 2 (2013): 79–87. https://doi.org/10.3109/09553002.2012.721052.
78. Slatkin, D. N., H. Blattmann, H. P. Wagner, M. A. Glotzer, and J. A. Laissue. '"Prospects for Microbeam Radiation Therapy of Brain Tumours in Children"'. Developmental Medicine and Child Neurology 51, no. 2 (2009): 163. https://doi.org/10.1111/j.1469-8749.2008.03187.x.
79. Slatkin, D. N., F. A. Dilmanian, M. M. Nawrocky, P. Spanne, J.-O. Gebbers, D. W. Archer, and J. A. Laissue. 'Design of a Multislit, Variable Width Collimator for Microplanar Beam Radiotherapy'. Review of Scientific Instruments 66, no. 2 (1 February 1995): 1459–60. https://doi.org/10.1063/1.1145940.
80. Slatkin, D N, P Spanne, F A Dilmanian, J O Gebbers, and J A Laissue. 'Subacute Neuropathological Effects of Microplanar Beams of X-Rays from a Synchrotron Wiggler'. Proceedings of the National Academy of Sciences of the United States of America 92, no. 19 (1995): 8783–8787. https://doi.org/10.1073/pnas.92.19.8783.
81. Smilowitz, H. M., H. Blattmann, E. Bräuer-Krisch, A. Bravin, M. Di Michiel, J. O. Gebbers, a. L. Hanson, et al. 'Synergy of Gene-Mediated Immunoprophylaxis and Microbeam Radiation Therapy for Advanced Intracerebral Rat 9L Gliosarcomas'. Journal of Neuro-Oncology 78, no. 2 (2006): 135–143. https://doi.org/10.1007/s11060-005-9094-9.
82. Smith, Richard W, Jiaxi Wang, Elisabeth Schültke, Colin B Seymour, Elke Bräuer-Krisch, Jean A Laissue, Hans Blattmann, and Carmel E Mothersill. 'Proteomic Changes in the Rat Brain Induced by Homogenous Irradiation and by the Bystander Effect Resulting from High Energy Synchrotron X-Ray Microbeams'. International Journal of Radiation Biology 89, no. 2 (2013): 118–127. https://doi.org/10.3109/09553002.2013.732252.
83. Smith, Richard, Jiaxi Wang, Colin Seymour, Cristian Fernandez-Palomo, Jennifer Fazzari, Elisabeth Schültke, Elke Bräuer-Krisch, Jean Laissue, Christian Schroll, and Carmel Mothersill. 'Homogenous and Microbeam X-Ray Radiation Induces Proteomic Changes in the Brains of Irradiated Rats and in the Brains of Nonirradiated Cage Mate Rats'. Dose-Response 16, no. 1 (January 2018): 155932581775006. https://doi.org/10.1177/1559325817750068.
84. Smyth, Lloyd M.L., Jacqueline F Donoghue, Jessica A Ventura, Jayde Livingstone, Tracy Bailey, Liam R.J. Day, Jeffrey C Crosbie, and Peter A.W. Rogers. 'Comparative Toxicity of Synchrotron and Conventional Radiation Therapy Based on Total and Partial Body Irradiation in a Murine Model'. Scientific Reports 8, no. 1 (2018): 12044. https://doi.org/10.1038/s41598-018-30543-1.
85. Smyth, Lloyd M.L., Sashendra Senthi, Jeffrey C Crosbie, and Peter A.W. Rogers. 'The Normal Tissue Effects of Microbeam Radiotherapy: What Do We Know, and What Do We Need to Know to Plan a Human Clinical Trial?' International Journal of Radiation Biology 92, no. 6 (2016): 302–311. https://doi.org/10.3109/09553002.2016.1154217.
86. Sprung, Carl N, Yuqing Yang, Helen B Forrester, Jason Li, Marina Zaitseva, Leonie Cann, Tina Restall, Robin L Anderson, Jeffrey C Crosbie, and Peter A. W. Rogers. 'Genome-Wide Transcription Responses to Synchrotron Microbeam Radiotherapy'. Radiation Research 178, no. 4 (2012): 249. https://doi.org/10.1667/rr2885.1.
87. Studer, F, R Serduc, B Pouyatos, T Chabrol, E. Bräuer-Krisch, M Donzelli, C Nemoz, J A Laissue, F. Estève, and A Depaulis. 'Synchrotron X-Ray Microbeams: A Promising Tool for Drug-Resistant Epilepsy Treatment'. Physica Medica 31, no. 6 (2015): 607–614. https://doi.org/10.1016/j.ejmp.2015.04.005.
88. Thomlinson, Bill. 'Medical Applications of Synchrotron Radiation'. Synchrotron Radiation News 24, no. 2 (2011): 2. https://doi.org/10.1080/08940886.2011.567156.
89. Thomlinson, W, P Berkvens, G Berruyer, B. Bertrand, H Blattmann, E Brauer-Krisch, T Brochard, et al. 'Research at the European Synchrotron Radiation Facility Medical Beamline.' Cellular and Molecular Biology (Noisy-Le-Grand, France) 46, no. 6 (2000): 1053–1063.
90. Uyama, Atsushi, Takeshi Kondoh, Nobuteru Nariyama, Keiji Umetani, Manabu Fukumoto, Kunio Shinohara, and Eiji Kohmura. 'A Narrow Microbeam Is More Effective for Tumor Growth Suppression than a Wide Microbeam: An in Vivo Study Using Implanted Human Glioma Cells'. Journal of Synchrotron Radiation 18, no. 4 (2011): 671–678. https://doi.org/10.1107/S090904951101185X.
91. Van Der Sanden, Boudewijn, Elke Bräuer-Krisch, Erik Albert Siegbahn, Clément Ricard, Jean Claude Vial, and Jean Laissue. 'Tolerance of Arteries to Microplanar X-Ray Beams'. International Journal of Radiation Oncology Biology Physics 77, no. 5 (2010): 1545–1552. https://doi.org/10.1016/j.ijrobp.2010.02.019.
92. Ventura, Jessica, Pavel N Lobachevsky, Jason S Palazzolo, Helen Forrester, Nicole M Haynes, Alesia Ivashkevich, Andrew W Stevenson, et al. 'Localized Synchrotron Irradiation of Mouse Skin Induces

Persistent Systemic Genotoxic and Immune Responses'. Cancer Research 77, no. 22 (2017): 6389–6399. https://doi.org/10.1158/0008-5472.CAN-17-1066.
93. Wang, S, and X Qian. 'Microbeam Radiation Therapy: A Review'. International Journal of Computational Biology and Drug Design 8, no. 2 (2015): 127–138.
94. Yang, Yuqing, Agnieszka Swierczak, Mohammad Ibahim, Premila Paiva, Leonie Cann, Andrew W. Stevenson, Jeffrey C. Crosbie, Robin L. Anderson, and Peter A.W. Rogers. 'Synchrotron Microbeam Radiotherapy Evokes a Different Early Tumor Immunomodulatory Response to Conventional Radiotherapy in EMT6.5 Mammary Tumors'. Radiotherapy and Oncology 133 (2019): 93–99. https://doi.org/10.1016/j.radonc.2019.01.006.
95. Zhong, Nan, Gerard M Morris, Tigran Bacarian, Eliot M Rosen, and F. Avraham Dilmanian. 'Response of Rat Skin to High-Dose Unidirectional X-Ray Microbeams: A Histological Study'. Radiation Research 160, no. 2 (2003): 133–142. https://doi.org/10.1667/3033.
